# Community-Engaged Research With Indigenous Communities to Improve Elder Health and Well-Being

**DOI:** 10.1093/geroni/igab046.894

**Published:** 2021-12-17

**Authors:** Jordan Lewis

## Abstract

Much of the past research conducted with tribal communities was coined "helicopter research," because researchers would enter the community, gather data, and leave the community, never to inform communities how the data was used or published, creating mistrust. Community Based Participatory Research (CBPR) is a research approach conducted as an equal partnership between community members, organizational representatives, and researchers that serve as guidelines for researchers working collaboratively with communities. This symposium will offer a panel of presentations highlighting research studies with tribal communities that honor and respect tribal sovereignty in addressing health and wellbeing among their older adults. The panel presentations will consist of presentations on dementia caregiving, generativity and successful aging, social support and diabetes management, elder-centered research methods.

